# Institutional Variance in Mortality after Percutaneous Coronary Intervention for Acute Myocardial Infarction in Korea, Japan, and Taiwan

**DOI:** 10.34172/ijhpm.2023.6796

**Published:** 2023-04-05

**Authors:** Hayato Yamana, Seyune Lee, Yi-Chieh Lin, Nan-He Yoon, Kiyohide Fushimi, Hideo Yasunaga, Shou-Hsia Cheng, Hongsoo Kim

**Affiliations:** ^1^Data Science Center, Jichi Medical University, Shimotsuke, Japan; ^2^Department of Clinical Epidemiology and Health Economics, School of Public Health, The University of Tokyo, Tokyo, Japan; ^3^Institute of Health and Environment, Seoul National University, Seoul, South Korea; ^4^Department of Healthcare Administration, College of Medicine, I-Shou University, Kaohsiung, Taiwan; ^5^Division of Social Welfare and Health Administration, Wonkwang University, Iksan, South Korea; ^6^Department of Health Policy and Informatics, Tokyo Medical and Dental University Graduate School, Tokyo, Japan; ^7^Institute of Health Policy and Management, College of Public Health, National Taiwan University, Taipei, Taiwan; ^8^Department of Public Health Sciences, Graduate School of Public Health, Seoul National University, Seoul, South Korea; ^9^Institute of Aging, Seoul National University, Seoul, South Korea; ^10^SOCIUM - Research Center on Inequality and Social Policy, University of Bremen, Bremen, Germany

**Keywords:** Acute Myocardial Infarction, Administrative Data, East Asia, Hospital Performance

## Abstract

**Background:** Although there have been studies that compared outcomes of patients with acute myocardial infarction (AMI) across countries, little focus has been placed on institutional variance of outcomes. The aim of the present study was to compare institutional variance in mortality following percutaneous coronary intervention (PCI) for AMI and factors explaining this variance across different health systems.

**Methods:** Data on inpatients who underwent PCI for AMI in 2016 were obtained from the National Health Insurance Data Sharing Service in Korea, the Diagnosis Procedure Combination (DPC) Study Group Database in Japan, and the National Health Insurance Research Database (NHIRD) in Taiwan. Multilevel analyses with inpatient mortality as the outcome and the hierarchical structure of patients nested within hospitals were conducted, adjusting for common patient-level and hospital-level variables. We compared the intraclass correlation coefficient (ICC) and the proportion of variance explained by hospital-level characteristics across the three health systems.

**Results:** There were 17 351 patients from 160 Korean hospitals, 29 804 patients from 660 Japanese hospitals, and 10 863 patients from 104 Taiwanese hospitals included in the analysis. Inpatient mortality rates were 6.3%, 7.3%, and 6.0% in Korea, Japan, and Taiwan, respectively. After adjusting for patient and hospital characteristics, Taiwan had the lowest variation in mortality (ICC, 1.8%), followed by Korea (2.2%) and then Japan (4.5%). The measured hospital characteristics explained 38%, 19%, and 9% of the institutional variance in Korea, Taiwan, and Japan, respectively.

**Conclusion:** Korea, Japan, and Taiwan had similarly uniform outcomes across hospitals for patients undergoing PCI for AMI. However, Japan had a relatively large institutional variance in mortality and a lower proportion of variation explainable by hospital characteristics, compared with Korea and Taiwan.

## Background

**Key Messages**

** Implications for policy makers**

- Three high-income economies with similar health systems had similarly small institutional variance in mortality of patients receiving percutaneous coronary intervention (PCI) for acute myocardial infarction (AMI).
- PCI for AMI was less centralized, and various procedures were performed in Japan compared with Korea and Taiwan.
- Further research to explain the variance may contribute to improvements in the delivery of care under different health systems.

** Implications for the public**

Patients should be provided with care of similar quality under one health system, regardless of where they are treated. This comparative study examined institutional difference in mortality following percutaneous coronary intervention (PCI) for acute myocardial infarction (AMI) in Korea, Japan, and Taiwan. Under the similar health systems characterized by universal coverage through mandatory social health insurance, the performance of hospitals performing PCI for AMI was uniformly good in a similar manner in Korea, Japan, and Taiwan. However, Japanese hospitals had relatively large variance in mortality. Further research on the mechanism of variation may improve the delivery of care.

Acute myocardial infarction (AMI) is a major cause of mortality worldwide.^1^ Extensive clinical trials have been conducted to update the treatment of AMI.^2-5^ In addition to advances in treatment, growing interest has been placed on the evaluation of healthcare systems, with the aim of optimizing the delivery of care.^6,7^ The mortality of patients with AMI, and especially of those who underwent percutaneous coronary intervention (PCI), are commonly used for the evaluation of institutions.^8,9^

 Several studies have compared the outcomes of patients with AMI across countries.^10-12^ A study using the national registries of Sweden and the United Kingdom found lower case mix-adjusted mortality in Sweden.^10^ The European Health Care Outcomes, Performance and Efficiency study, conducted in seven countries, showed a cross-country difference in age/sex-adjusted mortality.^11^ The European Health Care Outcomes, Performance and Efficiency study also reported an association between regional or hospital-level characteristics and mortality.^12^ These international benchmarking studies have provided insight that may lead to the improvement of healthcare systems.

 Two recent international comparative studies have additionally focused on institutional variance in the outcomes of patients with AMI.^13,14^ Such comparison of the variance across different health systems may contribute to improving healthcare equity. South Korea (hereafter Korea), Japan, and Taiwan are high-income economies in East Asia whose healthcare systems share several common characteristics such as universal coverage and mandatory social health insurance. The degree of institutional variance in outcomes has not been compared in such contexts. Additionally, it remains unclear whether such variance can be explained by hospital characteristics in a common manner.

 We conducted a retrospective observational study using data from Korea, Japan, and Taiwan to evaluate the institutional variance in outcomes following PCI for AMI. Patient-level data from representative administrative databases were used for the analysis, and we applied a multilevel analysis approach to quantify the levels of variance.

## Methods

###  Study Overview

 This was a retrospective study using National Health Insurance Data Sharing Service of the National Health Insurance Services (NHIS) in Korea, the Diagnosis Procedure Combination (DPC) Study Group Database in Japan, and National Health Insurance Research Database (NHIRD) by the Health and Welfare Data Science Center in Taiwan. Investigators in Korea, Japan, and Taiwan analyzed the patient-level data in each database separately using same protocol and analytic codes.

###  Data Source

 Details of the three databases have been provided elsewhere.^15-17^ The NHIS database in Korea and the NHIRD database in Taiwan store administrative claims data for virtually all residents. In Japan, the DPC-based payment system has been widely adopted by acute-care hospitals. The DPC Study Group Database is a nationwide inpatient database with data from approximately 1000 participating hospitals that use the DPC system, covering about 50% of all acute-care admissions in Japan. We also collected data on hospital and regional characteristics from the Survey of Medical Institutions, the 2014 Reporting System for Functions of Medical Institutions, Vital Statistics, and other publicly available data sources in Japan. Government statistics in 2016 were used to obtain data on hospital and regional characteristics in Taiwan. The NHIS database in Korea included hospital characteristics in 2016, and data on regional characteristics were obtained from government statistics.

###  Patients

 We identified patients who were admitted and discharged in 2016 with the primary diagnosis of AMI (*International Statistical Classification of Diseases and Related Health Problems, 10*^th^ *Edition [ICD-10]* code, I21). We excluded patients who had been hospitalized for AMI within 1 year of admission and patients under 50 years of age. We analyzed data on patients who underwent PCI during hospitalization. These criteria were set to create a relatively homogeneous population that allowed for comparison of institutional variance in outcomes following PCI for AMI. Patients whose data could not be merged with the hospital characteristics were excluded. To stabilize the estimates, we further excluded patients admitted to hospitals with fewer than five cases.

###  Variables

 Age was categorized as 50-64, 65-74, 75-84, or ≥85 years. Using the procedure codes shown in Table S1 of Supplementary file 1, we classified PCI procedures performed during hospitalization as angioplasty, stenting, or both. The numbers and subcategorizations of secondary diagnoses differed across the databases. In Taiwan, up to four secondary diagnoses could be recorded. In Korea, there was no limit to the number of secondary diagnoses. In Japan, secondary diagnoses were subcategorized into “comorbidities present on admission” and “complications arising after admission,” with up to 10 diagnoses each. To ensure comparability, we analyzed the first four secondary diagnoses in the Korean data, and the four “comorbidities present on admission” were used in the Japanese data. Comorbidities were summarized with the Charlson comorbidity index (CCI) using *ICD-10* codes and the updated algorithm proposed by Quan et al.^18,19^ CCI was categorized as 0, 1, 2, 3, or ≥ 4. The outcome in this study was in-hospital mortality.

 We examined the following hospital characteristics: ownership (private or public), location (rural or urban), teaching status, number of hospital beds, and patient volume. Location was based on administrative region, secondary medical area, and medical region in Korea, Japan, and Taiwan, respectively, and was categorized as rural when the population density of the area was less than 500 inhabitants/km^2^.^20^ Number of beds was categorized as <300, 300-499, or ≥500. Patient volume was defined as the number of patients with AMI included in the study sample for each hospital and was categorized into hospital quartiles.

###  Statistical Analysis

 To quantify institutional variance while considering the individual- and institution-level characteristics, we applied the framework of multilevel analysis of individual heterogeneity and discriminatory accuracy described by Merlo et al.^21-23^ We considered the data to have a two-level hierarchical structure, with patients nested within hospitals. We analyzed the hospital-level variance in inpatient mortality, adjusting for patient-level and hospital-level characteristics. Following a previous study,^21^ we constructed three models. Model 1 (individual effects model) was a conventional logistic regression model including only the patient-level covariates. Model 2 (general contextual effects model) was a multilevel logistic regression model with patient-level covariates and random intercepts for hospitals. Model 3 (specific contextual effects model) was a multilevel logistic regression model with both patient-level and hospital-level covariates and random intercepts for hospitals. Analyses were conducted using SAS 9.4 (SAS Institute Inc, Cary, NC, USA). The *logistic* procedure was used for Model 1, and the *glimmix* procedure specifying a binomial distribution and logit link function was used for Models 2 and 3.

 We used the area under the receiver operating characteristic curve (AUC) as a measure of the discriminatory accuracy of the three models. We calculated the differences in the AUCs between Models 1 and 2 and between Models 2 and 3. We calculated the intraclass correlation coefficient (ICC) in Models 2 and 3 to quantify general contextual effects. The ICC was calculated on the basis of the latent response formulation of the model.^21,22^ We also evaluated the median odds ratio (OR) in Models 2 and 3 as a measure of heterogeneity.^21,22^ The proportional change in variance (PCV), the proportion of hospital variance explained by adding hospital-level characteristics, was calculated as the difference between the hospital-level variance in Models 2 and 3 divided by the hospital-level variance in Model 2.^21,22^ To quantify the specific contextual effects of hospital-level characteristics on individual inpatient mortality, we calculated the 80% interval odds ratio (IOR-80%)^24^ and the proportion of opposed odds ratios (POOR)^21,25^ in Model 3. IOR-80% and POOR summarize the ORs of random comparisons of exposed and nonexposed clusters. IOR-80% represents the distribution of the ORs, and POOR is the proportion of ORs opposite the overall OR.^21,22,24,25^ Finally, we estimated the predicted mortality of a reference patient (a man aged 50–64 years who underwent angioplasty, with CCI = 0) in each hospital using Models 2 and 3.

 We conducted several sensitivity analyses to confirm the robustness of the results. First, we excluded patients admitted to hospitals with fewer than 10 cases (instead of fewer than 5 cases as performed in the main analysis). Second, we used all secondary diagnoses in the Japanese and Korean data to calculate the CCI. Additional patient-level and hospital-level data were available in the Japanese dataset. We therefore conducted three additional analyses using the Japanese data. In one analysis, we added patient-level variables, and in another, we added hospital-level variables. The added variables and their categorization were as follows: Killip classification, Japan Coma Scale (0, 1–3, 10-30, or 100-300), body mass index (<18.5, 18.5-24.9, 25.0-29.9, or ≥30.0), the DPC category of the hospitals (1, 2, or 3), the number of ambulance transports per year (<3000 or ≥3000), and the number of angiography machines (≤2 and ≥3). Finally, we changed the definition of hospital volume from the number of PCI cases for AMI in our sample to all PCI cases according to the Japanese hospital-level data in June 2014. Patients with missing data on these additional variables were excluded from these sensitivity analyses.

 We conducted an auxiliary analysis to evaluate the representativeness of the Japanese DPC data. Using data from the Reporting System for Functions of Medical Institutions, hospitals included in the study were compared with other DPC hospitals and non-DPC hospitals in Japan that performed PCI in June 2014. The numbers of PCI cases in June 2014, hospital beds, and annual ambulance transports were compared.

## Results

**Figure 1:**
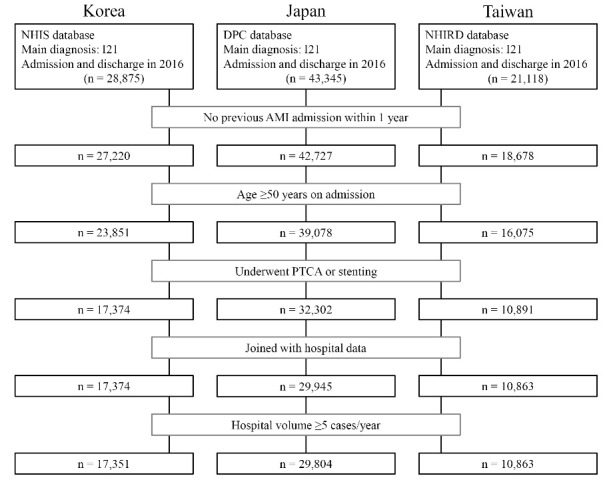
Selection of study patients who underwent percutaneous coronary intervention for acute myocardial infarction in Korea, Japan, and Taiwan. Abbreviations: AMI, acute myocardial infarction; DPC, Diagnosis Procedure Combination; NHIS, National Health Insurance Services; NHIRD, National Health Insurance Research Database; PTCA, percutaneous transluminal coronary angioplasty.

 The flow of patient selection is presented in Figure 1. There were 28 875, 43 345, and 21 118 patients initially identified in the Korean, Japanese, and Taiwanese databases, respectively. Analyses were conducted using data on 17 351 patients from 160 Korean hospitals, 29 804 patients from 660 Japanese hospitals, and 10 863 patients from 104 Taiwanese hospitals.

 Patient characteristics are presented in Table 1. Inpatient mortality rates were 6.3% in Korea, 7.3% in Japan, and 6.0% in Taiwan. The mean length of hospital stay (standard deviation) was 7.4 (8.0), 16.7 (13.8), and 7.1 (7.3) days in Korea, Japan, and Taiwan, respectively. Hospital characteristics are presented in Table 2. The quartile points of hospital volume were 37, 79, and 154 in Korea; 20, 37, and 62 in Japan; and 38, 79, and 148 in Taiwan.

**Table 1:** Characteristics of Patients With Acute Myocardial Infarction Undergoing Percutaneous Coronary Intervention in Korea, Japan, and Taiwan

**Characteristic**	**Korea (N = 17 351)**		**Japan (N = 29 804)**		**Taiwan (N = 10 863)**		***P *** **Value**
Gender							
Male	12402	(71)	22241	(75)	8181	(75)	<.001
Female	4949	(29)	7563	(25)	2682	(25)	
Age (y)							
Mean (SD)	68.0	(10.7)	71.1	(10.6)	68.0	(11.1)	
50–64	7306	(42)	8276	(28)	4659	(43)	<.001
65–74	4619	(27)	9886	(33)	2922	(27)	
75–84	4344	(25)	8215	(28)	2291	(21)	
≥85	1082	(6)	3427	(11)	991	(9)	
Procedure							
Angioplasty	1098	(6)	2335	(8)	1107	(10)	<.001
Stenting	15689	(90)	19257	(65)	8414	(77)	
Angioplasty and stenting	564	(3)	8212	(28)	1342	(12)	
CCI							
Mean (SD)	0.72	(1.07)	0.87	(1.12)	0.84	(1.40)	
0	11027	(64)	17033	(57)	6264	(58)	<.001
1	1450	(8)	2114	(7)	1352	(12)	
2	4002	(23)	9061	(30)	1878	(17)	
3	607	(3)	1034	(4)	968	(9)	
≥ 4	265	(2)	562	(2)	401	(4)	
Inpatient death	1086	(6)	2183	(7)	649	(6)	<.001

Abbreviations: SD, standard deviation; CCI, Charlson comorbidity index. Note: Data shown as No. (%) unless otherwise specified.

**Table 2:** Characteristics of Hospitals With Admission of Patients With Percutaneous Coronary Intervention for Acute Myocardial Infarction in Korea, Japan, and Taiwan

**Characteristic**	**Korea (N = 160)**		**Japan (N = 660)**		**Taiwan (N = 104)**		***P*** ** Value**
	**No.**	**(%)**	**No.**	**(%)**	**No.**	**(%)**	
Ownership							
Private	153	(96)	393	(60)	72	(69)	<.001
Public	7	(4)	267	(40)	32	(31)	
Location							
Rural	50	(31)	321	(49)	22	(21)	<.001
Urban	110	(69)	339	(51)	82	(79)	
Teaching status							
Teaching	55	(34)	77	(12)	96	(92)	<.001
Non-teaching	105	(66)	583	(88)	8	(8)	
Number of beds							
≤299	1	(1)	145	(22)	9	(9)	<.001
300–499	72	(45)	276	(42)	27	(26)	
≥500	87	(54)	239	(36)	68	(65)	
Hospital volume							
1st quartile	41	(26)	171	(26)	27	(26)	1.000
2nd quartile	39	(24)	165	(25)	25	(24)	
3rd quartile	40	(25)	159	(24)	26	(25)	
4th quartile	40	(25)	165	(25)	26	(25)	

 The results of the regression analysis in Models 1, 2, and 3 are presented in Tables S2-S4, and the specific contextual effects of hospital-level characteristics in Model 3 are presented in Table S5. The teaching status of the hospital was significantly associated with patient outcome in Taiwan but not in Korea or Japan. The number of hospital beds was associated with patient outcome in Taiwan and Korea but not in Japan.

**Figure 2:**
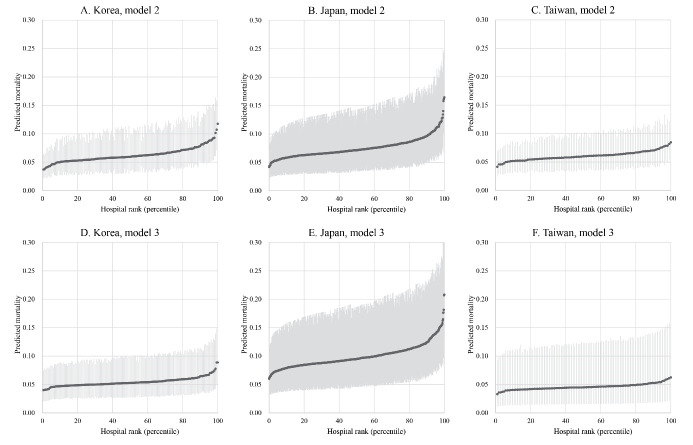
Predicted Mortality of a Reference Patient for Each Hospital in Korea, Japan, and Taiwan, using Model 2 and Model 3. Note: Model 2 a is multilevel logistic regression model with patient-level covariates and random intercepts for hospitals. Model 3 is a multilevel logistic regression model with patient-level and hospital-level covariates and random intercepts for hospitals. The reference patient is male, aged 50-64 years, underwent angioplasty, and has a CCI of 0.

Table 3 shows the summary statistics of the three models. The ICC was <10% in Korea, Japan, and Taiwan, indicating a low level of variation in outcomes at hospital level. In Model 3, Taiwan had the smallest variation (1.8%), followed by Korea (2.2%) and then Japan (4.5%). The median OR was largest in Japan (1.46), followed by Korea (1.30) and then Taiwan (1.26). The PCV was largest in Korea (38%), followed by Taiwan (19%) and then Japan (9%). The predicted mortality of the reference patient (a man aged 50–64 years who underwent angioplasty, with CCI = 0) for each hospital in Models 2 and 3 is presented in Figure 2, which visualizes the findings shown by the summary statistics.

**Table 3:** Summary Statistics of Models Predicting In-Hospital Mortality of Patients With Acute Myocardial Infarction in Korea, Japan, and Taiwan

**Statistic**	**Model**^a^	**Korea**	**Japan**	**Taiwan**
AUC	1	0.703	0.657	0.674
	2	0.735	0.715	0.697
	3	0.732	0.713	0.698
Difference in AUCs	2−1	0.033	0.058	0.023
	3−2	−0.003	−0.002	0.001
ICC (%)	2	3.54	4.99	2.22
	3	2.22	4.54	1.80
Median OR	2	1.39	1.49	1.30
	3	1.30	1.46	1.26
PCV (%)	3−2	38.2	9.4	19.3

Abbreviations: AUC, Area under the receiver operating characteristic curve; ICC, Intraclass correlation coefficient; OR, odds ratio; PCV, Proportional change in variance. ^a^Model 1, logistic regression with patient-level covariates; Model 2, multilevel logistic regression with patient-level covariates and random intercepts for hospitals; Model 3, multilevel logistic regression with patient-level and hospital-level covariates and random intercepts for hospitals.

 The summary statistics of the sensitivity analyses are shown in Tables S6-S8. Exclusion of hospitals with fewer than 10 cases or use of all secondary diagnoses had little impact on the overall results. The ICC increased and the PCV decreased when patient-level variables were added in the Japanese data (6.9% for Model 3 and 0.41%, respectively).

 The comparison of the Japanese hospitals included in this study with other DPC and non-DPC hospitals performing PCI is presented in Table S9. The hospitals in this study represented 50% of hospitals performing PCI in Japan, accounting for 61% of all PCI procedures in the country. The hospitals examined in this study were larger and had higher volumes of PCI compared with the hospitals that were not examined here.

## Discussion

 The present study was conducted using data from Korea, Japan, and Taiwan to evaluate the institutional variance in inpatient mortality following PCI for AMI. The analysis of a total of 58 018 patients from 924 hospitals using administrative databases showed small institutional variance within Korea, Japan, and Taiwan. Taiwan had the smallest variance among the three (ICC, 1.8%), followed by Korea (2.2%) and then Japan (4.5%). The measured hospital characteristics explained 38%, 19%, and 9% of the institutional variance in Korea, Taiwan, and Japan, respectively.

 We used comparative databases from Korea, Japan, and Taiwan. The NHIS database in Korea and the NHIRD database in Taiwan covered the entire population. Although the Japanese DPC database did not cover all hospitalizations in the country, our auxiliary analysis indicated that more than half of all cases in the country were included in these data. The background characteristics of patients were mostly similar across Korea, Japan, and Taiwan, except for the slightly older population in Japan.

 The average annual number of cases per hospital (180 in Korea, 66 in Japan, and 203 in Taiwan) and the quartile points for hospital volume showed that Japanese hospitals had a lower patient volume than Korean and Taiwanese hospitals. Additionally, Korean and Taiwanese hospitals had more beds than Japanese hospitals. Thus, PCI for AMI was more centralized in large hospitals in Korea and Taiwan than was the case in Japan. The Japanese hospitals examined in this study had a larger patient volume compared with the Japanese hospitals that were not examined here. There may be more small-volume hospitals performing PCI for AMI in Japan than was reflected in the data used in this study.

 Several previous studies have reported international comparisons of the outcomes of patients with AMI.^10-12^ However, few international comparative studies have focused on institutional variance in outcomes. The ICC, which measures the share of the total variance that is at the hospital level, was used as the main indicator of hospital-level variance. In Model 2, the ICC was relatively large in Japan (5.0%) compared with Korea (3.5%) and Taiwan (2.2%). However, Korea, Japan, and Taiwan all had small ICCs (<10%),^26^ showing similar outcomes across hospitals within each health system. Additionally, overall inpatient mortality was 6.3% in Korea, 7.3% in Japan, and 6.0% in Taiwan. Although patient backgrounds may differ, these numbers were similar to that reported for the Unites States.^27^ Thus, following the classification used in a previous study,^26^ we conclude that hospitals performed similarly well in Korea, Japan, and Taiwan. After further adjustment by the measured hospital characteristics, Taiwan had the smallest remaining variation (1.8%), followed by Korea (2.2%) and then Japan (4.5%).

 We also analyzed the associations between hospital characteristics and individual patient outcomes. The interpretation of contextual variables in multilevel analyses is difficult because a regression coefficient represents the cluster-specific effect but a contextual variable is constant for all individuals within a cluster. IOR-80% and POOR are summary measures of the ORs comparing the exposed and nonexposed clusters. IOR-80% represents the distribution of these ORs, and POOR (ranging from 0% to 50%) is the proportion of ORs opposite the overall OR.^21,22,24,25^ In this study, IOR-80% excluded one, and POOR was low for some hospital-level variables in Korea and Taiwan. However, for hospital-level variables in Japan, IOR-80% was wide and POOR was close to 50%. This indicates greater heterogeneity in hospital-level effects in Japan than in Korea and Taiwan. A smaller PCV in Japan compared with Korea and Taiwan also implies that institutional variance in Japan could not be explained by the measured characteristics. The sensitivity analysis including additional hospital-level variables in Japan resulted in only a small change in the PCV, which supports the robustness of the results.

 Korea, Japan, and Taiwan are three high-income economies in East Asia with similar health systems that are characterized by universal coverage through mandatory social health insurance. Previous studies have shown differences in the outcomes of patients with AMI between countries with different health systems.^10,11^ In contrast, in the three similar contexts examined in this study, the performance of hospitals performing PCI for AMI, measured by inpatient mortality, was uniformly good in a similar manner. However, we did observe some differences in institutional variance. Furthermore, the degree to which basic hospital characteristics could explain the variance differed across Korea, Japan, and Taiwan. Examining the detailed reasons for these differences was beyond the scope of this study. Nevertheless, we observed that PCI for AMI was less centralized and that various procedures were performed in Japan, compared with Korea and Taiwan. Such practice patterns may be an influencing factor. Further research to explain the variance may contribute to improvements in the delivery of care in these three health systems and in others.

 Several limitations of the present study must be acknowledged. First, a limited number of patient-level variables were available in the administrative databases. Importantly, ST-elevation and non-ST-elevation myocardial infarction could not be differentiated using ICD-10 codes. Residual clustering of patients may be included in the hospital-level variance. However, hospital-level variance was small in Korea, Japan, and Taiwan. Additionally, the sensitivity analysis including additional patient characteristics in Japan resulted in only a modest change in the ICC. Thus, absence of variables would have a small impact on the overall results. Our analysis showed that the PCI rates differed across Korea, Japan, and Taiwan. The addition of patient-level variables may allow for comparison of practice patterns in future studies. Second, the databases lacked physician-level variables such as physician volume and experience. Addition of these variables may further decrease the hospital-level variance. Third, we did not have data covering all Japanese hospitals. The inclusion of different types of hospitals might increase the hospital-level variance in Japan. Additionally, small differences among the databases, such as the number of diagnoses and definition of procedures, may have affected the comparison. Fourth, we evaluated in-hospital mortality because a longitudinal analysis was not possible using the Japanese data. Outcomes may be affected by differences in the length of stay and place of discharge. Further research is necessary to evaluate outcomes such as 30-day and 1-year mortality. Fifth, the analysis was performed using data of a single year. Analysis of different years may clarify the change in outcomes over time. Finally, we targeted a relatively homogeneous and describable population of patients undergoing PCI for AMI to improve the comparability. The results may not be generalizable to other quality indicators for AMI or other conditions.

## Conclusion

 Korea, Japan, and Taiwan had similarly uniform outcomes for patients undergoing PCI for AMI across hospitals. However, Japan had relatively large institutional variation and a lower proportion of variation that could be explained by hospital characteristics, compared with Korea and Taiwan. Further research on the mechanism of variation may lead to improvements in the delivery of care.

## Ethical issues

 The study was approved by the Institutional review board of Seoul National University, Seoul, South Korea. Because this was a secondary analysis of anonymized data, the need for informed consent was waived.

## Competing interests

 Authors declare that they have no competing interests.

## Authors’ contributions

 Conceptualization: Hongsoo Kim, Hayato Yamana.

 Data curation: Hongsoo Kim, Shou-Hsia Cheng, Kiyohide Fushimi, Hideo Yasunaga.

 Formal analysis: Hayato Yamana, Seyune Lee, Nan-He Yoon, Yi-Chieh Lin, Shou-Hsia Cheng, Hongsoo Kim.

 Funding acquisition: Hongsoo Kim, Kiyohide Fushimi, Hideo Yasunaga, Shou-Hsia Cheng.

 Project administration: Hongsoo Kim, Seyune Lee.

 Supervision: Hongsoo Kim, Shou-Hsia Cheng, Kiyohide Fushimi, Hideo Yasunaga.

 Writing–original draft: Hayato Yamana.

 Writing–review & editing: Shou-Hsia Cheng, Hongsoo Kim, Seyune Lee, Nan-He Yoon, Yi-Chieh Lin, Kiyohide Fushimi, Hideo Yasunaga.

## Funding

 AXA research fund (900-20170006), The Ministry of Health, Labour and Welfare, Japan (21AA2007 and 22AA2003) and the Ministry of Education, Culture, Sports, Science and Technology, Japan (20H03907).

## Supplementary files


Supplementary file 1 contains Tables S1-S9.
