# Supplementary material for: Institutional Variance in Mortality after Percutaneous Coronary Intervention for Acute Myocardial Infarction in Korea, Japan, and Taiwan
Source: Int J Health Policy Manag. 2023 Apr 5;12:6796. doi: 10.34172/ijhpm.2023.6796 (PMC10461903; doi:10.34172/ijhpm.2023.6796)
Supplement: Supplementary file 1 — contains Tables S1-S9. [file ijhpm-12-6796-s001.pdf]

Article title: Institutional Variance in Mortality after Percutaneous Coronary Intervention for Acute Myocardial Infarction in Korea, Japan, and Taiwan

Journal name: International Journal of Health Policy and Management (IJHPM)

Authors' information: Hayato Yamana[1,2], Seyune Lee[3], Yi-Chieh Lin[4], Nan-He Yoon[5], Kiyohide Fushimi[6], Hideo Yasunaga[2], Shou-Hsia Cheng[7], Hongsoo Kim[3,8,9,10]*

[1]Data Science Center, Jichi Medical University, Shimotsuke, Japan.

[2]Department of Clinical Epidemiology and Health Economics, School of Public Health, The University of Tokyo, Tokyo, Japan.

[3]Institute of Health and Environment, Seoul National University, Seoul, South Korea.

[4]Department of Healthcare Administration, College of Medicine, I-Shou University, Kaohsiung, Taiwan.

[5]Division of Social Welfare and Health Administration, Wonkwang University, Iksan, South Korea.

[6]Department of Health Policy and Informatics, Tokyo Medical and Dental University Graduate School, Tokyo, Japan.

[7]Institute of Health Policy and Management, College of Public Health, National Taiwan University, Taipei, Taiwan.

[8]Department of Public Health Sciences, Graduate School of Public Health, Seoul National University, Seoul, South Korea.

[9]Institute of Aging, Seoul National University, Seoul, South Korea.

[10]SOCIUM - Research Center on Inequality and Social Policy, University of Bremen, Bremen, Germany

(*Corresponding author: hk65@snu.ac.kr)

Supplementary file 1 contains Tables S1-S9.

Table S1. Codes used to identify percutaneous coronary intervention procedures from databases in Korea, Japan, and Taiwan

Procedure	Korea	Japan	Taiwan
Angioplasty	M6551	K546	02704ZZ
	M6552	K547	3E07317
		K548	02713ZZ
			02714ZZ
			02723ZZ
			02724ZZ
			02733ZZ
			02734ZZ
Stenting	M6561	K549	027034Z
	M6562		02703DZ
	M6563		027044Z
	M6564		02704DZ
			027134Z
			02713DZ
			027144Z
			02714DZ
			027234Z
			02723DZ
			027244Z
			02724DZ
			027334Z
			02733DZ
			027344Z
			02734DZ
			0270346
			02703D6
			0270446
			02704D6
			0271346
			02713D6
			0271446
			02714D6
			0272346
			02723D6
			0272446
			02724D6
			0273346
			02733D6
			0273446
			02734D6

Table S2. Results of logistic regression analysis of in-hospital mortality (Model 1)

Variable	Korea			Japan			Taiwan		
	OR	(95% CI)	*p*	OR	(95% CI)	*p*	OR	(95% CI)	*p*
Sex									
Female	1.10	(0.96, 1.26)	.185	1.01	(0.91, 1.11)	.894	1.23	(1.03, 1.47)	.021
Age, years									
65–74	2.30	(1.90, 2.78)	< .001	1.28	(1.11, 1.47)	< .001	1.63	(1.29, 2.06)	< .001
75–84	3.69	(3.07, 4.43)	< .001	2.31	(2.02, 2.65)	< .001	3.02	(2.42, 3.79)	< .001
≥ 85	7.28	(5.81, 9.13)	< .001	4.39	(3.78, 5.09)	< .001	4.94	(3.83, 6.38)	< .001
Procedure									
Stenting	0.36	(0.30, 0.43)	< .001	0.50	(0.44, 0.57)	< .001	0.51	(0.41, 0.63)	< .001
Angioplasty and stenting	0.57	(0.40, 0.80)	.001	0.49	(0.42, 0.57)	< .001	0.62	(0.46, 0.83)	.002
Charlson comorbidity index									
1	1.07	(0.86, 1.32)	.558	1.06	(0.90, 1.26)	.492	0.76	(0.59, 0.98)	.035
2	1.03	(0.89, 1.20)	.675	0.90	(0.81, 0.99)	.037	0.86	(0.70, 1.07)	.173
3	1.24	(0.92, 1.68)	.158	1.43	(1.16, 1.76)	< .001	0.43	(0.31, 0.61)	< .001
≥ 4	1.42	(0.92, 2.20)	.110	1.31	(1.01, 1.71)	.045	0.81	(0.54, 1.21)	.298

Abbreviations: CI, confidence interval; OR, odds ratio

Table S3. Results of multilevel analysis of in-hospital mortality (Model 2)

Variable	Korea			Japan			Taiwan		
	OR	(95% CI)	*p*	OR	(95% CI)	*p*	OR	(95% CI)	*p*
Sex									
Female	1.11	(0.97, 1.27)	.143	1.00	(0.91, 1.11)	.955	1.23	(1.03, 1.47)	.023
Age, years									
65–74	2.30	(1.90, 2.79)	< .001	1.28	(1.11, 1.47)	< .001	1.65	(1.31, 2.09)	< .001
75–84	3.64	(3.03, 4.38)	< .001	2.34	(2.04, 2.68)	< .001	3.07	(2.45, 3.85)	< .001
≥ 85	7.13	(5.67, 8.95)	< .001	4.50	(3.87, 5.23)	< .001	5.01	(3.88, 6.48)	< .001
Procedure									
Stenting	0.36	(0.30, 0.43)	< .001	0.50	(0.43, 0.57)	< .001	0.49	(0.39, 0.61)	< .001
Angioplasty and stenting	0.57	(0.40, 0.80)	.001	0.47	(0.40, 0.55)	< .001	0.62	(0.46, 0.84)	.002
Charlson comorbidity index									
1	1.08	(0.87, 1.34)	.499	1.05	(0.89, 1.25)	.543	0.77	(0.59, 0.99)	.041
2	1.01	(0.86, 1.18)	.885	0.90	(0.81, 1.00)	.047	0.86	(0.69, 1.06)	.158
3	1.25	(0.92, 1.69)	.159	1.43	(1.16, 1.77)	< .001	0.43	(0.30, 0.60)	< .001
≥ 4	1.39	(0.90, 2.15)	.143	1.31	(1.00, 1.72)	.048	0.81	(0.54, 1.21)	.300

Abbreviations: CI, confidence interval; OR, odds ratio

Table S4. Results of multilevel analysis of in-hospital mortality with hospital-level variables (Model 3)

Variable	Korea			Japan			Taiwan		
	OR	(95% CI)	*p*	OR	(95% CI)	*p*	OR	(95% CI)	*p*
Sex									
Female	1.11	(0.96, 1.27)	.147	1.01	(0.91, 1.11)	.913	1.23	(1.03, 1.47)	.024
Age, years									
65–74	2.31	(1.91, 2.79)	< .001	1.28	(1.11, 1.47)	< .001	1.65	(1.30, 2.08)	< .001
75–84	3.66	(3.05, 4.41)	< .001	2.34	(2.04, 2.68)	< .001	3.07	(2.45, 3.85)	< .001
≥ 85	7.10	(5.65, 8.91)	< .001	4.51	(3.88, 5.24)	< .001	4.98	(3.85, 6.44)	< .001
Procedure									
Stenting	0.36	(0.30, 0.43)	< .001	0.50	(0.44, 0.58)	< .001	0.49	(0.39, 0.62)	< .001
Angioplasty and stenting	0.57	(0.40, 0.80)	.001	0.47	(0.40, 0.55)	< .001	0.63	(0.47, 0.85)	.002
Charlson comorbidity index									
1	1.07	(0.86, 1.33)	.540	1.05	(0.88, 1.24)	.607	0.76	(0.59, 0.98)	.034
2	1.03	(0.88, 1.21)	.717	0.89	(0.80, 0.99)	.035	0.85	(0.69, 1.06)	.152
3	1.27	(0.94, 1.73)	.125	1.41	(1.14, 1.74)	.001	0.42	(0.30, 0.60)	< .001
≥ 4	1.41	(0.91, 2.19)	.122	1.30	(0.99, 1.70)	.058	0.82	(0.54, 1.23)	.331
Public hospital	1.17	(0.78, 1.75)	.450	1.06	(0.94, 1.20)	.337	0.94	(0.75, 1.19)	.620
Urban hospital	1.08	(0.89, 1.31)	.440	1.01	(0.90, 1.14)	.880	1.06	(0.82, 1.38)	.668
Non-teaching hospital	0.78	(0.65, 0.94)	.009	0.79	(0.66, 0.94)	.010	0.45	(0.21, 0.99)	.050
Hospital beds									
300–499	1.66	(1.21, 2.29)	.002	0.92	(0.77, 1.10)	.357	1.64	(0.70, 3.82)	.256
≥ 500	1.72	(1.22, 2.42)	.002	1.01	(0.83, 1.22)	.959	1.94	(0.78, 4.81)	.156
Patient volume									
2nd quartile	0.94	(0.66, 1.35)	.746	1.00	(0.81, 1.25)	.967	0.73	(0.47, 1.15)	.179
3rd quartile	0.84	(0.58, 1.22)	.364	0.87	(0.71, 1.08)	.212	0.62	(0.39, 1.00)	.051
4th quartile	0.63	(0.42, 0.95)	.028	0.86	(0.70, 1.07)	.184	0.64	(0.40, 1.02)	.064

Abbreviations: CI, confidence interval; OR, odds ratio

Table S5. Specific contextual effects of hospital-level characteristics in multilevel analysis of in-hospital mortality (Model 3)

Variable	Korea		Japan		Taiwan	
	IOR-80%	POOR	IOR-80%	POOR	IOR-80%	POOR
Public hospital	(0.71,1.92)	34	(0.52,2.18)	46	(0.60,1.47)	43
Urban hospital	(0.66,1.77)	42	(0.49,2.07)	49	(0.68,1.65)	43
Non-teaching hospital	(0.47,1.28)	26	(0.38,1.61)	33	(0.29,0.70)	1
Hospital beds						
300–499	(1.01,2.73)	9	(0.45,1.88)	44	(1.05,2.56)	8
≥ 500	(1.05,2.82)	8	(0.49,2.06)	50	(1.24,3.03)	3
Patient volume						
2nd quartile	(0.57,1.55)	44	(0.49,2.06)	50	(0.47,1.14)	19
3rd quartile	(0.51,1.38)	33	(0.43,1.79)	40	(0.40,0.97)	8
4th quartile	(0.39,1.04)	12	(0.42,1.77)	40	(0.41,1.00)	10

Abbreviations: IOR-80%, 80% interval odds ratio; POOR, proportion of opposed odds ratios

Table S6. Summary statistics of models predicting in-hospital mortality of patients with acute myocardial infarction in Korea, Japan, and Taiwan (sensitivity analysis excluding hospitals with fewer than 10 cases)

Statistic	Model†	Korea	Japan	Taiwan
Area under the receiver operating characteristic curve	1	0.702	0.656	0.674
	2	0.735	0.714	0.697
	3	0.732	0.713	0.698
Difference in areas under the receiver operating characteristic curves	2−1	0.033	0.058	0.023
	3−2	−0.003	−0.002	0.001
Intraclass correlation coefficient (%)	2	3.55	5.03	2.24
	3	2.23	4.55	1.71
Median odds ratio	2	1.39	1.49	1.30
	3	1.30	1.46	1.26
Proportional change in variance (%)	3−2	37.9	9.8	24.0
Number of analyzed patients		17,290	29,419	10,825
Number of analyzed hospitals		152	604	98

†Model 1, logistic regression with patient-level covariates; Model 2, multilevel logistic regression with patient-level covariates and random intercepts for hospitals; Model 3, multilevel logistic regression with patient-level and hospital-level covariates and random intercepts for hospitals

Table S7. Summary statistics of models predicting in-hospital mortality of patients with acute myocardial infarction in Korea and Japan (sensitivity analysis using all secondary diagnoses)

Statistic	Model†	Korea	Japan
Area under the receiver operating characteristic curve	1	0.709	0.660
	2	0.740	0.716
	3	0.739	0.714
Difference in areas under the receiver operating characteristic curves	2−1	0.031	0.056
	3−2	−0.001	−0.002
Intraclass correlation coefficient (%)	2	3.65	5.00
	3	2.39	4.54
Median odds ratio	2	1.40	1.49
	3	1.31	1.46
Proportional change in variance (%)	3−2	35.4	9.6

†Model 1, logistic regression with patient-level covariates; Model 2, multilevel logistic regression with patient-level covariates and random intercepts for hospitals; Model 3, multilevel logistic regression with patient-level and hospital-level covariates and random intercepts for hospitals

Table S8. Summary statistics of additional models predicting in-hospital mortality of patients with acute myocardial infarction in Japan

Statistic	Model†	Patient-level variables added	Hospital-level variables added	Hospital volume using all PCI
Area under the receiver operating characteristic curve	1	0.887	0.658	0.658
	2	0.902	0.713	0.717
	3	0.902	0.712	0.716
Difference in areas under the receiver operating characteristic curves	2−1	0.016	0.056	0.059
	3−2	0.000	−0.001	−0.001
Intraclass correlation coefficient (%)	2	6.95	4.90	5.05
	3	6.93	4.46	4.79
Median odds ratio	2	1.60	1.48	1.49
	3	1.60	1.45	1.47
Proportional change in variance (%)	3−2	0.41	9.33	5.43
Number of analyzed patients		26,736	29,169	28,954
Number of analyzed hospitals		659	630	643

†Model 1, logistic regression with patient-level covariates; Model 2, multilevel logistic regression with patient-level covariates and random intercepts for hospitals; Model 3, multilevel logistic regression with patient-level and hospital-level covariates and random intercepts for hospitals
Abbreviation: PCI, percutaneous coronary intervention

Table S9. Comparison of Japanese hospitals included in this study with other DPC and non-DPC hospitals performing PCI in June 2014

Characteristic	DPC hospitals examined	DPC hospitals not examined	Non-DPC hospitals not examined
Number of hospitals	603[†]	391	210
PCI cases in each hospital, median [IQR]	16 [8,25]	9 [4,19]	6 [2,13]
Total number of PCI cases	11,712	5527	2084
Number of hospital beds, median [IQR]	410 [303,574]	320 [230,417]	148 [88,212]
Annual ambulance transports, median [IQR]	2904 [1906,4526]	2378 [1326,3591]	691 [305,1259]

[†]Excludes 57 hospitals not performing PCI in June 2014.
Abbreviations: DPC, Diagnosis Procedure Combination database; PCI, percutaneous coronary intervention; IQR interquartile range
